# Visual Scanning and Falls in Older Adults: The Mexico Health and Aging Study

**DOI:** 10.1111/jgs.70051

**Published:** 2025-09-01

**Authors:** Angela L. Xu, Melissa Li, Anoopum S. Gupta, Susanne M. Morton, Ali G. Hamedani

**Affiliations:** ^1^ Department of Neurology, Perelman School of Medicine University of Pennsylvania Philadelphia Pennsylvania USA; ^2^ Department of Ophthalmology, Perelman School of Medicine University of Pennsylvania Philadelphia Pennsylvania USA; ^3^ Department of Epidemiology, Perelman School of Medicine University of Pennsylvania Philadelphia Pennsylvania USA; ^4^ Department of Neurology, Massachusetts General Hospital Harvard Medical School Boston Massachusetts USA; ^5^ Department of Physical Therapy University of Delaware Newark Delaware USA; ^6^ Center for Clinical Epidemiology and Biostatistics, Perelman School of Medicine University of Pennsylvania Philadelphia Pennsylvania USA; ^7^ Leonard Davis Institute of Health Economics University of Pennsylvania Philadelphia Pennsylvania USA

**Keywords:** falls, ocular mobility, visual scanning

## Abstract

**Background:**

For selected patients at increased fall risk, physical therapy may include instruction to look around and observe the environment to identify obstacles, known as visual scanning or tracking, and avoid them. Whether visual scanning reduces fall risk more broadly in the general population is unknown.

**Methods:**

Using data from the Mexico Health and Aging Study (MHAS), a longitudinal, nationally representative study of adults 50 years of age and older in Mexico (*n* = 13,850), we measured the association between visual scanning test performance and three fall‐related outcomes: any fall in the previous 2 years, recurrent falls, and falls with injury. We conducted both cross‐sectional and longitudinal analyses over 3 years of follow‐up; logistic regression models were adjusted for demographic variables, self‐reported comorbidities, daily activity limitations, and a standardized, composite measure of attention (Z‐score of summed backwards counting and serial 7's tests) using inverse probability weighting. Inverse probability weights were multiplied by MHAS survey weight to account for complex survey design.

**Results:**

Visual scanning ability within the lowest quartile was associated with an increased risk of recurrent falls over 3 years of follow‐up (adjusted OR 1.36, 95% CI: 1.02–1.80). Associations with incident falls and falls with injury attenuated after covariate adjustment.

**Conclusions:**

People with lower levels of visual scanning behavior were more likely to suffer recurrent falls over time. This highlights the importance of visual function and behavior in mitigating fall risk and suggests that efforts to improve visual scanning may help to prevent recurrent falls in older adults.

## Graphical abstract

Cross‐sectional association between visual scanning and fall‐related outcomes in the Mexican Health and Aging Study (MHAS). Unadjusted odds ratio (OR) and 95% confidence intervals (CI) represented using orange squares and lines, and adjusted OR and 95% CI represented using blue circles and lines. Logistic regression models adjusted for age, gender, marital status, rural location, education level, smoking, hypertension, diabetes, heart disease, stroke, arthritis, cancer, fractures, visual impairment, urinary urgency, pain, dementia, depressive symptoms, lower extremity functional limitation, ADL limitations, and attention span using inverse probability weighting.

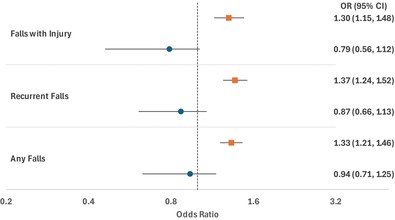

**Summary**

- Key points
  ○ Falls are a leading cause of morbidity and mortality worldwide, and efforts to reduce fall risk take a multimodal approach.
  ○ Visual scanning training has been used in physiotherapy for patients with prior mobility or visual impairment to reduce fall risk; whether this intervention can apply more broadly to the wider population is unknown.
  ○ Using a large, population‐based health survey, we found that poor visual scanning ability was associated with a higher risk of recurrent falls over time in the general population.
- Why does this paper matter?
  ○ Our findings suggest that improving visual scanning can reduce the risk of recurrent falls in older adults, which is a major cause of morbidity and mortality.

## 1 Introduction

Falls are the second leading cause of unintentional death worldwide, and over 80% of fall‐related deaths occur in low‐ and middle‐income countries [1]. In physical therapy, one strategy to reduce fall risk is visual scanning, where a patient examines their physical environment quickly to avoid collision with objects. However, whether visual scanning is associated with fall risk in the general population is unknown. The objective of this study was to characterize the association between visual scanning abilities and falls using data from a large, population‐based Mexican health survey.

## 2 Methods

### 2.1 Standard Protocol Approvals and Patient Consents

This study was reviewed as exempt from approval by the University of Pennsylvania Institutional Review Board. Informed consent was previously obtained at study enrollment.

### 2.2 Study Population

We used data from the Mexico Health and Aging Study (MHAS), a longitudinal population‐based survey of adults over 50 living in Mexico that has been ongoing since 2001. A full description of MHAS's sampling and study procedure can be found elsewhere [2, 3, 4]. Briefly, MHAS utilizes a nationally representative sample of the Mexican population, urban and rural, via direct, in‐person interviews with subjects and/or their spouses. For this analysis, we used data from waves 4 (2015) and 5 (2018), during which visual scanning was measured as described below.

### 2.3 Primary Outcomes and Exposures

In each wave, subjects were asked if they had fallen in the last 2 years, how many falls they suffered, and if they had ever been injured from a fall. We defined recurrent falls as two or more falls within a given timeframe, consistent with previous research [5]. Visual scanning was measured by showing participants a target shape and then presenting another test sheet filled with dozens of irregularly spaced figures (including the prompt image). Subjects were instructed to identify and circle as many figures as possible that matched the prompt image in 1 min, with a maximum score of 60 [6]. We compared individuals in the bottom quartile of visual scanning to those in the top three quartiles. Since visual scanning also depends upon attention and concentration, we also examined performance on backward counting and serial 7's.

### 2.4 Statistical Analysis

For cross‐sectional analyses, we used logistic regression models to measure the association between visual scanning at wave 4 and fall outcomes at wave 4. For longitudinal analyses, we measured the association between visual scanning at wave 4 and incident fall outcomes in wave 5. To adjust for confounding, we created a propensity score model of visual scanning as a function of the following covariates: age; gender; marital status; rural location; education; smoking; diagnoses of hypertension, diabetes, heart disease, stroke, cancer, and arthritis; visual impairment; urinary urgency; chronic pain; dementia; depressive symptoms; lower extremity functional limitations; ADL limitations; and a standardized, composite measure of attention (Z‐score of summed backwards counting and serial 7's tests). Lower extremity functional limitations were quantified using a sum of self‐reported inability to do any of the following (Range: 0–9): walk across a room, walk one block, walk several blocks, sit for 2 h, get up from a chair, climb one flight of stairs, climb several flights of stairs, kneel or crouch, or push or pull large objects. ADL limitations included self‐reported ability to dress, eat, bathe, get out of bed, and toilet (Range: 0–5). We then calculated inverse probability weights and multiplied them by the MHAS survey weight for analysis. Statistical analysis was performed using STATA version 17 (College Station, TX) and statistical significance was defined at the *p* < 0.05 level.

## 3 Results

There were 14,769 participants in MHAS wave 4, of whom 929 were excluded because they were proxy respondents and therefore lacked data on visual scanning and other performance measures. Of the remaining 13,840 participants, 3739 (25.1%) had reduced visual scanning test performance, and the baseline characteristics of this cohort are presented in Table 1.

**TABLE 1:** Baseline characteristics of subjects with or without recent fall history.

	Any fall in last 2 years	
	Yes (*n* = 6124)	No (*n* = 7716)
Age in years (mean, SD)	64.7 (10.0)	62.8 (9.6)
Visual scanning score (mean, SD)	28.0 (16.5)	31.4 (16.5)
Lowest quartile visual scanning (*n*)	1905	1832
Female (*n*, %)	4058 (65.1%)	4057 (50.3%)
Married (*n*, %)	3930 (64.7%)	5516 (71.8%)
Rural (*n*, %)	1865 (37.2%)	2183 (34.8%)
Education		
Illiterate	913 (15.7%)	888 (12.5%)
Less than lower secondary	4455 (72.6%)	5451 (70.2%)
Upper secondary and vocational	201 (3.9%)	326 (4.5%)
Tertiary	494 (7.9%)	958 (12.9%)
Ever smoked (*n*, %)	2322 (39.9%)	3195 (42.4%)
Hypertension (*n*, %)	3886 (58.3%)	4158 (49.5%)
Diabetes (*n*, %)	1840 (27.2%)	1895 (22.4%)
Heart disease (*n*, %)	641 (9.3%)	577 (5.7%)
Stroke (*n*, %)	267 (4.3%)	237 (2.7%)
Arthritis (*n*, %)	1983 (30.0%)	1758 (18.8%)
Cancer (*n*, %)	287 (4.5%)	238 (2.1%)
Fractures (*n*, %)	1718 (26.4%)	1274 (14.3%)
Visual impairment (*n*, %)	5550 (90.6%)	6802 (88.0%)
Urinary urgency (*n*, %)	1620 (23.4%)	1183 (13.0%)
Pain (*n*, %)	2919 (49.0%)	2393 (31.1%)
Dementia (*n*, %)	496 (7.4%)	348 (3.7%)
Depressive symptoms (*n*, %)	2376 (40.2%)	1897 (24.4%)
Lower extremity functional limitations (mean, SD)	4.0 (2.5)	3.3 (2.6)
ADL limitations (mean, SD)	0.5 (1.0)	0.3 (0.9)
Attention test Z‐score (mean, SD)	−0.1 (1.0)	0.0 (1.0)

**FIGURE 1:**
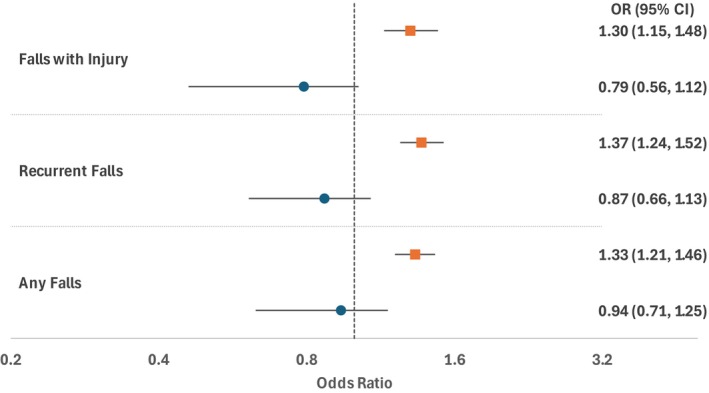
Cross‐sectional association between visual scanning and fall outcomes. *Unadjusted odds ratio (OR) and 95% confidence intervals (CI) represented using orange squares and lines, and adjusted OR and 95% CI represented using blue circles and lines. Adjusted for age, gender, marital status, rural location, education level, smoking, hypertension, diabetes, heart disease, stroke, arthritis, cancer, fractures, visual impairment, urinary urgency, pain, dementia, depressive symptoms, lower extremity functional limitation, ADL limitations, and attention span using inverse probability weighting.

**FIGURE 2:**
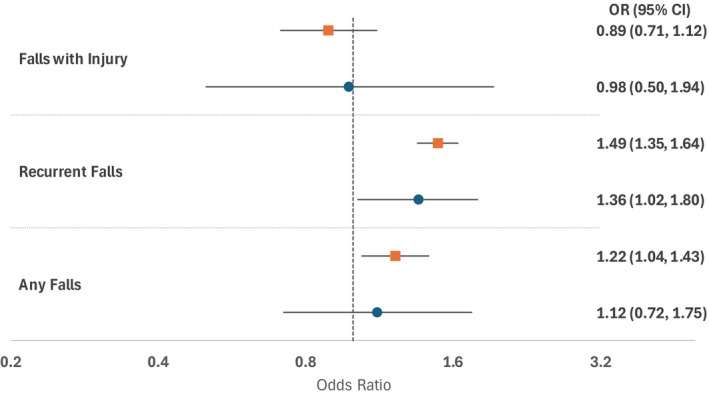
Longitudinal association between visual scanning and fall outcomes. *Unadjusted odds ratio (OR) and 95% confidence intervals (CI) represented using orange squares and lines, and adjusted OR and 95% CI represented using blue circles and lines. Adjusted for age, gender, marital status, rural location, education level, smoking, hypertension, diabetes, heart disease, stroke, arthritis, cancer, fractures, visual impairment, urinary urgency, pain, dementia, depressive symptoms, lower extremity functional limitation, ADL limitations, and attention span using inverse probability weighting.

Cross‐sectional and longitudinal associations between visual scanning and fall outcomes are presented in Figures 1 and 2, respectively. In unadjusted analyses, individuals in the bottom quartile of visual scanning at wave 4 were more likely to report a history of falls in wave 4 (Figure 1) and incident falls in wave 5 (Figure 2). Although many of these associations attenuated after covariate adjustment, reduced visual scanning remained significantly associated with recurrent falls at wave 5 in our adjusted longitudinal analyses (Figure 2).

## 4 Discussion

In this study, people with lower levels of visual scanning behavior were more likely to suffer recurrent falls over time. This suggests that visual function and behavior, including ocular motor and saccadic ability, alongside cognitive function, may contribute to fall risk. Efforts to improve visual scanning may reduce the risk of recurrent falls in older adults.

Although visual scanning was associated with fall recurrence, we did not observe an association for all falls or falls with injury. This may be because falls are common in this population [1], so a single fall may be nonspecific and less reflective of an underlying state of increased risk. Other limitations include the self‐reporting of falls, lack of data on other visual measures such as visual acuity and contrast sensitivity, and the possibility of type 1 error. Previous studies have shown that recurrent fallers are at a higher risk of functional decline, morbidity, and mortality [7] than both non‐fallers and single‐fallers, which supports the idea of fall recurrence as a distinct outcome whose risk factors may differ from other fall‐related outcomes. Efforts to improve visual scanning have been implemented as treatments for poststroke visual field defects [8], but our results suggest that these rehabilitation strategies may be more broadly applicable to the older adult population. Because visual scanning is also reflective of deeper cognitive factors [9], it may be an accessible marker for the underlying neurocognitive function that is important to maintain for fall prevention [10].

## Author Contributions

Angela L. Xu was responsible for statistical analysis and primarily drafted and edited the manuscript. Melissa Li, Anoopum S. Gupta, and Susanne M. Morton assisted in the review and editing of the manuscript. Ali G. Hamedani conceived of the study and critically reviewed the manuscript. Ali G. Hamedani had full access to the data and affirms that all authors contributed significantly to the work.

## Disclosure

The sponsor played no role in the design or execution of this project.

## Conflicts of Interest

The authors declare no conflicts of interest.

## Data Availability

Data files and documentation are public use and available at www.MHASweb.org.
